# BsR1, a broad-spectrum antibacterial peptide with potential for plant protection

**DOI:** 10.1128/spectrum.02578-23

**Published:** 2023-11-10

**Authors:** Pei Song, Li Zhao, Li Zhu, Gan Sha, Wubei Dong

**Affiliations:** 1 Department of Plant Pathology, College of Plant Science and Technology and the Key Lab of Crop Disease Monitoring & Safety Control in Hubei Province, Huazhong Agricultural University, Wuhan, China; University of California, San Diego, La Jolla, California, USA

**Keywords:** antimicrobial peptide, antibacterial drug, antifungal gene, broad-spectrum resistance, cell membrane interrupting peptide

## Abstract

The urgent need for new antibacterial drugs arises from the emergence of bacterial multidrug resistance due to antibiotic overuse. Antimicrobial peptides, crucial components of innate immunity in animals and plants, exhibit effectiveness against multidrug-resistant bacteria while minimizing the development of drug resistance. In this study, the antibacterial peptide BsR1, comprising 21 amino acids, was finalized by predicting the active site of an antifungal gene and sequence optimization. BsR1 displayed broad-spectrum antibacterial activities against diverse Gram-positive and Gram-negative bacteria, with a low minimum inhibitory concentration of 4.25–17 μM. Stability experiments demonstrated that BsR1 has high resistance to thermal, ultraviolet, and acid-base conditions, while revealing increased sensitivity to divalent ions Ca^2+^ and Mg^2+^. The mode of action of BsR1 involved cell membrane damage, leading to bacterial cell structure disruption and subsequent death. Secondary structure prediction indicated a linear helical conformation with a positive charge of +7.5, facilitating its interaction with the target cell membrane. BsR1 exhibited excellent biological safety, as it did not induce necrosis in tobacco leaves, and the low observed hemolytic effect on mammalian cells with a value of 3.26%. Additionally, BsR1 demonstrated the ability to enhance disease resistance in rice and effectively curbed the spread of rice bacterial blight. This research presents BsR1 as a novel approach and potential medication in the development of antibacterial drugs, offering a valuable tool in combating pathogenic microorganisms, particularly in plants.

**IMPORTANCE:**

This study addresses the critical need for new antibacterial drugs in the face of bacterial multidrug resistance resulting from antibiotic overuse. It highlights the significance of antimicrobial peptides as essential components of innate immunity in animals and plants, which have been proven effective against multidrug-resistant bacteria and are difficult to develop resistance against. This study successfully synthesizes a broad-spectrum antibacterial peptide, BsR1, with strong inhibitory activities against various Gram-positive and Gram-negative bacteria. BsR1 demonstrates favorable stability and a mode of action that damages bacterial cell membranes, leading to cell death. It also exhibits biological safety and shows potential in enhancing disease resistance in rice. This research offers a novel approach and potential medication for antibacterial drug development, presenting a valuable tool in combating pathogenic microorganisms, particularly in plants.

## INTRODUCTION

The presence of pathogenic microorganisms poses a persistent challenge to agricultural development, resulting in substantial global economic losses. In recent times, focused investigations and widespread utilization of targeted antimicrobial drugs have been pursued. Nevertheless, the excessive utilization of antibiotics has fostered the emergence of multidrug resistance among pathogenic microorganisms. Consequently, the exploration of alternative approaches, such as antimicrobial peptides, has gained prominence as a potential solution to counter this predicament (1, 2). Antimicrobial peptides (AMPs) represent a class of small molecular polypeptides consisting of 10–60 amino acids and are integral components of the innate immunity found in animals and plants (3 – 5). Termed host defense peptides, AMPs have garnered significant attention and are cataloged in the antimicrobial peptide database (APD3), the most extensive repository of antimicrobial peptides, comprising 3,569 peptides from diverse life kingdoms, including those synthesized through predictive approaches. Antimicrobial peptides, as a type of polypeptide, have a secondary structure consistent with that of non-antimicrobial peptides: α-helix (e.g., LL-37 and Magainin-2) (6, 7), β-sheet (e.g., Tachyplesin I) (8), a combination of α-helix and β-sheet (e.g., Thionin) (9), and structures lacking α-helix and β-sheet elements (e.g., indolicidin) (10). Generally, AMPs exhibit a positive charge due to the presence of lysine, arginine, and histidine residues, coupled with hydrophobicity derived from tryptophan, phenylalanine, leucine, and isoleucine residues. These amino acid compositions confer the basic positive charge and hydrophobic characteristics observed in AMPs (11). There are currently four hypotheses regarding the antibacterial effect of antimicrobial peptides on microorganisms. The first hypothesis is the “barrel-stave model,” which suggests that antimicrobial peptides embed in phospholipid bilayers to form a round barrel with peptides as the stave (12), eventually forming a channel in the cell membrane through which the cell contents flow out. The second hypothesis is the “carpet model,” which proposes that antimicrobial peptide molecules gather on one side of the cell membrane surface and cover the surface of the cell membrane like a carpet, resulting in the formation of a potential difference between the inside and outside of the cell membrane (13 – 15). This causes the fluidity of the phospholipid bilayer to become disordered, which leads to its collapse and cracking. The third hypothesis is the “toroidal-pore model,” which suggests that the antimicrobial peptide is adsorbed to the cell membrane due to electrostatic interaction. The peptide aggregation causes the lipid head group region to contact the hydrophilic part of the peptide, which leads to the bending of the phospholipid bilayer to form a ring, ultimately destroying its integrity (16 – 18). The fourth hypothesis is the “aggregate channel model,” which proposes that the aggregation channel model will randomly form channels in any direction on the cell membrane. This model includes peptide-lipid interactions and peptide-ion interactions. These ions exist on the surface of the cell membrane, and antimicrobial peptides can compete with them for membrane binding, ultimately destabilizing membrane stability (19 – 21). Antimicrobial peptides are known to have various mechanisms of action, including their positive charge which allows them to interact with negatively charged cell membranes. This interaction disrupts the fluidity of the membrane and ultimately leads to cell lysis. Apart from this, these peptides can also interact with microbial DNA (22), RNA (23), and proteins (24); inhibit bacterial biofilm formation (25); interact with ribosomes (26); and even inhibit bacterial swarmming and swimming (27).

Recent research has shown that synthetic antimicrobial peptides (SAMPs) are gaining popularity over natural antimicrobial peptides (NAMPs) due to their superior structure, mechanism of action, toxicity, and ability to overcome drug resistance (28, 29). NAMPs are often limited by natural disadvantages such as susceptibility to protease degradation, high toxicity, and low yield. However, SAMPs that are rationally designed and modified can overcome these limitations and significantly improve antibacterial activity. For example, AamAP1-Lysine (a SAMP) has an inhibitory concentration range of 5–7.5 μM, while AamAP1 (a NAMP) has an inhibitory concentration range of 20–150 μM (30). These findings highlight the potential of modified antimicrobial peptides to enhance antibacterial activity. In the rational design of antimicrobial peptides, increasing the positive charge is an important factor. For instance, MSI-99, a synthetic derivative of Magainin II, has more positive charges than the parent peptide, which increases its antibacterial activity by two to five times *in vitro* (31). Similarly, BP-76, a modified version of Pep3, is less hemolytic (32). Replacing the first amino acid of a peptide chain with glycine can effectively prevent cleavage by aminopeptidase (33, 34). The N- and C-terminal regions of antimicrobial peptides are crucial for their antibacterial activity. The N-terminal interacts with the microbial membrane or substances in the membrane, while the C-terminal participates in the transmembrane transport of the antimicrobial peptide and the interaction with the biomembrane (35, 36). Therefore, designing the N- and C-terminal regions of antimicrobial peptides can enhance their stability (37), increase antibacterial activity (38, 39), and reduce hemolytic activity (40, 41). Common modification methods include N-terminal acetylation (CH_3_CO−) and C-terminal amidation (−NH_2_) (42).

Bioinformatics prediction is a crucial aspect for designing antimicrobial peptides and is widely used by researchers. Classic antimicrobial peptide databases and prediction sites like CAMP (Collection of Anti-Microbial Peptides) (43, 44) and ClassAMP (45) offer researchers a great deal of convenience. Further research on the active area of antimicrobial peptides is possible with secondary structure prediction websites like C-I-TASSER (46) and PEP-FOLD3 (47). The establishment and use of computational models such as SVM (support vector machine), RF (random forest), and ANN (artificial neural network) has led to a significant improvement in the prediction and development of antimicrobial peptides. In this study, the active site of the existing antifungal gene *Fg*Mt1 (48) was analyzed and predicted using bioinformatics. The amino acids at key sites were replaced accordingly, and the final sequence was chemically synthesized for application and follow-up studies on antibacterial activity and disease resistance. In prior research, a peptide named Mt1, comprising 80 amino acids, was discovered with the capability to target the branched-chain amino acid biosynthesis/metabolism pathway. This action demonstrated inhibition of the normal growth, development, and pathogenicity of wild-type *Fusarium graminearum*. Due to the inherent challenges associated with in vitro synthesis and the lethality for microbial expression of the 80-amino acid peptide, an alternative approach was adopted. Bioinformatics analysis was employed to forecast the modification of this polypeptide, utilizing Mt1 as a template. The objective was to transform it into an antimicrobial peptide, and subsequently, an exploration was conducted into its antimicrobial and antibacterial activities, along with its potential in combating diseases.

## RESULTS

### The source and design of antimicrobial peptide BsR1

The antimicrobial peptide BsR1 was designed based on the sequence motif derived from the antifungal gene *Fg*Mt1. Structure prediction analysis revealed multiple potential active regions within *Fg*Mt1 that could be converted into antimicrobial peptides. To determine the N-terminus and C-terminus of BsR1, we utilized the AntiBP Server, an antibacterial peptide prediction website. The complete amino acid sequence of *Fg*Mt1 (excluding the first Methionine) was submitted to the website, and three algorithm models [artificial neural network, quantitative matrices (QM), and support vector machine] were employed to predict the likelihood of different segments of this polypeptide functioning as antimicrobial peptides. Table 1 displays the two sequences with the highest probabilities for each terminal according to the SVM and QM algorithms, selecting the most favorable N-terminus and C-terminus sequences. Analysis based on SVM and QM algorithms indicated that the polypeptide sequences starting at positions 2 and 43 in the N-terminal, as well as positions 58 and 65 in the C-terminal, displayed a high probability of becoming antimicrobial peptides. By combining these identified start sites, the middle segment spanning from the second position of the N-terminal to the 58th position of the C-terminal exhibited the highest probability of becoming an antimicrobial peptide, represented by the sequence FVCRYHRQKWRTRGREWLRRE (Table 2). Statistical analysis conducted by Lata et al. suggests that glycine and cysteine are preferred at the N-terminus and C-terminus of antimicrobial peptides, respectively (49). Consequently, the initial “F” and “E” residues of the N-terminus and C-terminus were replaced with “G” and “C,” respectively, resulting in the final sequence of BsR1 as GVCRYHRQKWRTRGREWLRRC.

**TABLE 1:** Prediction of antimicrobial peptides BsR1’s active regions^*a*^

Peptide			Start position	Score	Antibacterial activity
SVM	N-terminal	FVCRYHRQKWRTRGR	2	2.384	Yes
		CRYHRQKWRTRGREW	4	0.759	Yes
	C-terminal	ERRLWERGRTRWKQR	58	0.515	Yes
		GRTRWKQRHYRCVFP	65	0.447	Yes
ANN	N-terminal	PFVCRYHRQKWRTRG	1	1	Yes
		RSVLFAQETAPELSL	24	1	Yes
	C-terminal	AQRNLSLEPATEQAF	38	0.98	Yes
		ASLCFAQRNLSLEPA	33	0.97	Yes
QM	N-terminal	FCLSALLRSPCSGSF	43	1.232	Yes
		FVCRYHRQKWRTRGR	2	1.116	Yes
	C-terminal	GRTRWKQRHYRCVFP	65	1.242	Yes
		SGSCPSRLLASLCFA	24	0.662	Yes

^*a*^ The peptides’ length is 15 amino acids, and the start position is counted from both the N-terminal and C-terminal of the *Fg*Mt1 sequence.

**TABLE 2:** Predicted probability of the combined sequence becoming an antimicrobial peptide^*a*^

Peptide			Start position	Score	Antibacterial activity
SVM	N-terminal	FVCRYHRQKWRTRGR	1	2.384	Yes
	C-terminal	ERRLWERGRTRWKQR	1	0.515	Yes
ANN	N-terminal	FVCRYHRQKWRTRGR	1	1	Yes
	C-terminal	ERRLWERGRTRWKQR	1	1	Yes
QM	N-terminal	FVCRYHRQKWRTRGR	1	1.116	Yes
	C-terminal	ERRLWERGRTRWKQR	1	0.37	Yes

^*a*^ The peptides’ length is 15 amino acids, and the start position is counted from both the N-terminal and C-terminal of the *Fg*Mt1 sequence.

**Fig 1:**
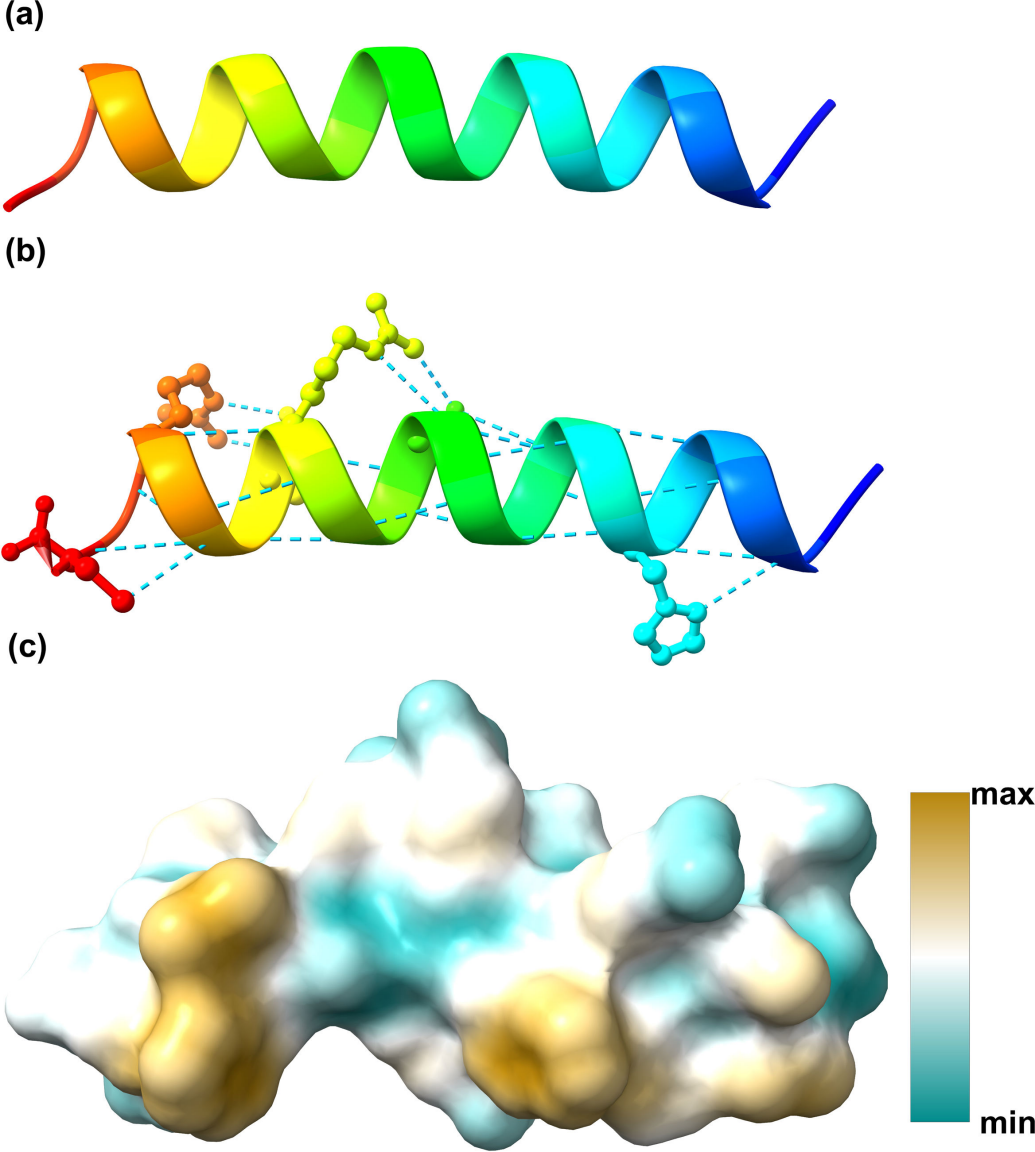
Secondary structure and hydrophobicity analysis of BsR1. (a) Predicted secondary structure of BsR1 peptide using the RPBS website. (b) Representation of the 22 potential hydrogen bonds in BsR1, indicated by blue dashed lines, as predicted by the RPBS website. (c) Hydrophobicity analysis of BsR1 surface using the RPBS website. The color scale ranges from blue to brown, indicating increasing hydrophobicity values. The structures presented above were visualized in the PDB format of BsR1 using Chimera X software.

In this study, we conducted a comprehensive analysis of the properties and structure of BsR1, a 21-amino acid cationic peptide. Using the APD and ressource parisienne en bioinformatique structurale (RPBS), we determined that BsR1 carries a net charge of +7.25 and exhibits a stable secondary structure with 22 hydrogen bonds, forming an α-helix (Fig. 1a and b). Figure S1a illustrates a high proportion of positively charged and hydrophobic amino acids, with arginine being prominently present (Fig. S1b). The molecular weight of BsR1 is calculated as 2803.283, and it demonstrates significant hydrophobicity, with a total hydrophobic ratio of 29% and a Wimley-White whole-residue hydrophobicity of 3.98. Effective antimicrobial peptides require membrane affinity and transmembrane activity. According to predictions from the RPBS website, these peptides exhibit hydrophobic amino acids on the same plane, with higher hydrophobicity regions concentrated on the same surface. This arrangement enhances the likelihood of contact with the cell membrane and facilitates transmembrane activity. BsR1, an arginine-rich cationic antimicrobial peptide, is predicted to possess membrane-binding ability and exert its action on the cell membrane. To confirm this, we chemically synthesized BsR1 through GenScript Company. Additionally, we synthesized another polypeptide, BsR2 (see Fig. S2 for details), derived from *Fg*Mt1, which lacks apparent secondary structure characteristics, serving as a control for antibacterial experiments. Both peptides were dissolved in sterile water following synthesis. Please refer to the supplementary file for further details regarding the synthesis process.

### BsR1 has specific antibacterial activity against bacteria

**Fig 2:**
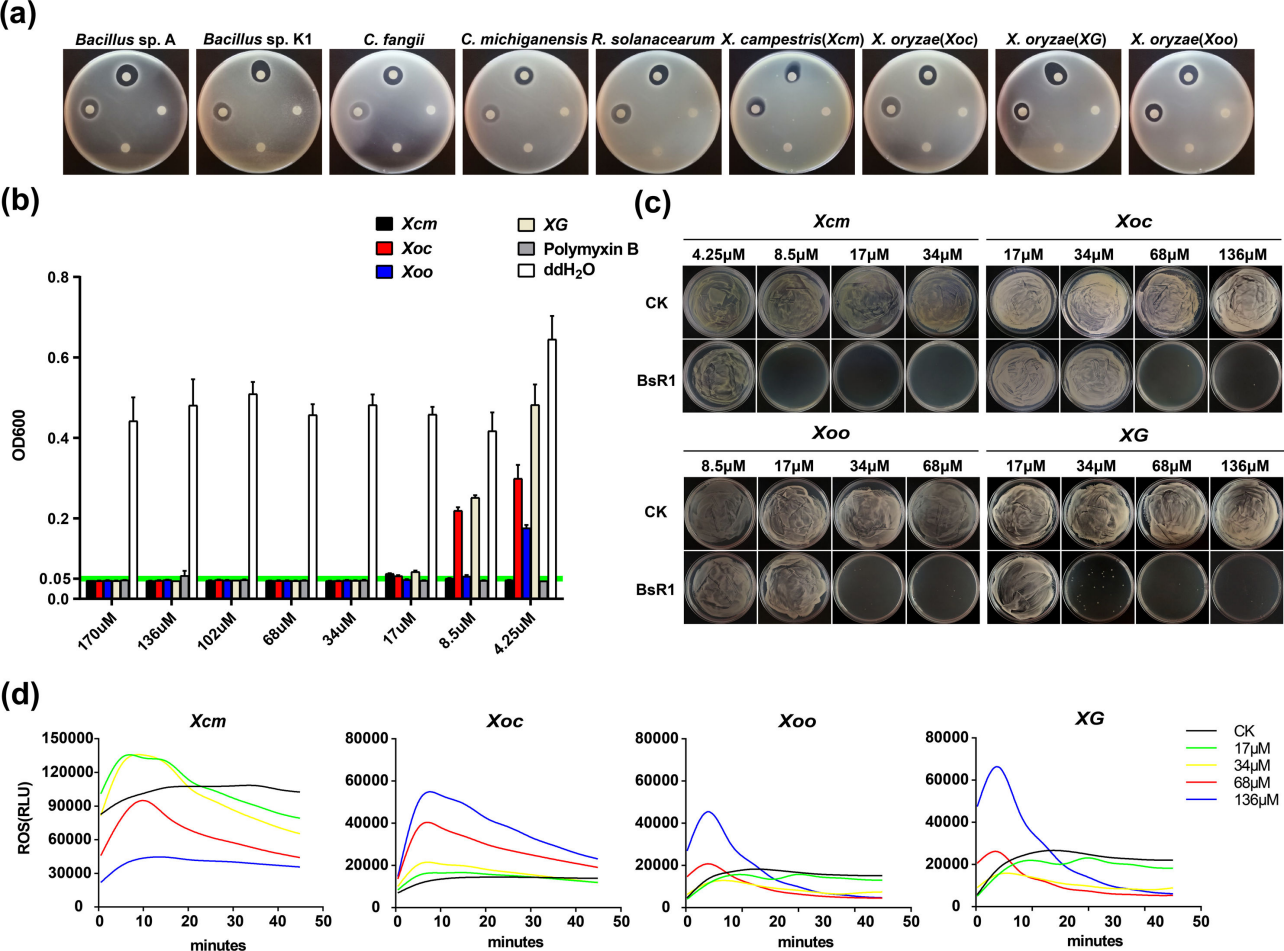
Broad-spectrum antibacterial activity of the antimicrobial peptide BsR1. (a) Plate layout: the upper sample hole on each plate served as a positive control containing polymyxin B, while the lower sample hole served as a negative control containing ddH_2_O. The left sample hole was used for BsR1, and the right sample hole was used for BsR2 as a peptide control. For each sample hole, dropwise add 20 µL of sample (BsR1/ddH_2_O/polymyxin B). Each experiment was independently repeated three times with three replicates each time. (b) Minimum inhibitory concentration (MIC) determination: The MIC value of BsR1 against the four tested bacteria was determined by observing the concentration near or below the optical density at 600 nm (OD_600_) = 0.05 standard line, indicating inhibitory concentration. The experiment included a positive control of polymyxin B and a negative control of ddH_2_O for all strains tested. Polymyxin B was used at a concentration of 0.2 mg/mL. Each experiment was independently repeated three times with three replicates each time. The error bar represents the standard error of measurement (SEM). (c) Minimum bactericidal concentration (MBC) determination: BsR1 was employed to determine the MBC of the four tested bacterial strains. Plates with a colony count exceeding five were considered non-MBC concentrations, while ddH_2_O served as the negative control (CK). Each experiment was independently repeated three times with three replicates each time. (d) ROS burst measurement: following treatment with BsR1, the level of ROS burst was measured in the four tested bacteria. ddH_2_O was used as the negative control (CK). Each experiment was independently repeated three times, with three replicates each time.

BsR1 was tested using plate antibacterial assays (see Fig. 2a) against a total of nine plant pathogenic and non-pathogenic bacteria (refer to Materials and Methods?). In each plate, BsR1 was applied to the leftmost sample well, while BsR2 was placed in the rightmost sample well. The top well contained polymyxin B as a positive control, while the bottom well contained sterile water as a negative control. The results revealed that BsR1 exhibited antibacterial activity against all nine tested Gram-negative and Gram-positive bacteria. The presence of the zone of inhibition indicated the inhibitory activity of BsR1 against cultured strains of bacteria. Notably, the four bacteria that displayed the specific antibacterial activity were *Xcm*, *Xoc*, *Xoo*, and *XG*, which belong to the Gram-negative *Xanthomonas* species. Consequently, these four bacteria were selected as the focus of further investigation to elucidate the antibacterial mechanism of BsR1.

The minimum inhibitory concentration (MIC) is a critical parameter that reflects the bacteriostatic ability of antimicrobial peptides. In our study, an optical density (OD) at 600 nm (OD_600_) value below 0.05 was considered the MIC concentration. The experimental results demonstrated the MIC values of BsR1 against the four strains as follows: MIC(*Xcm*) = 4.25 µM, MIC(*Xoc*) = 17 µM, MIC(*Xoo*) =8 .5 µM, and MIC(*XG*) =1 7 µM (see Fig. 2b). Another important indicator of antimicrobial activity is the minimum bactericidal concentration (MBC). MBC values were determined by spreading the MIC test samples on lysogeny broth (LB) plates and considering concentrations where fewer than five single colonies were observed as the MBC value. The experimental results revealed the corresponding MBC values for BsR1 against the four indicator bacteria: MBC(*Xcm*) = 8.5 µM, MBC(*Xoc*) = 68 µM, MBC(*Xoo*) = 34 µM, and MBC(*XG*) = 68 µM (see Fig. 2c).

Based on these findings, it can be inferred that BsR1 exhibits specific antibacterial activity against Gram-negative bacteria. The lowest MIC value observed was 4.25 µM, and the lowest MBC value recorded was 8.5 µM. To assess whether antimicrobial peptides induce oxidative stress responses in microorganisms, we treated the four indicator bacteria with different concentrations of BsR1 and measured the levels of reactive oxygen species (ROS). The ROS content indicated whether the antimicrobial peptide could trigger the accumulation of ROS, posing a threat to bacterial cells (refer to Fig. 2d). Among *Xoc*, *Xoo*, and *XG*, the ROS level was highest at the highest concentration of BsR1, peaked at 5–10 minutes, and decreased rapidly over time. In the case of *Xcm*, the higher the concentration of BsR1, the lower the level of active oxygen. At a concentration of 136 µM, the peak value was the lowest. At a concentration of 17 µM, the peak level of active oxygen in the bacteria was the highest, with a longer duration of decline.

Considering the MIC values of the four tested strains, it is speculated that *Xcm* has the lowest MIC value. It is possible that when bacterial cells are exposed to high concentrations of antimicrobial peptides, the cells rapidly undergo lysis without the opportunity to produce oxidative stress. Therefore, it can be inferred that BsR1 does not primarily destroy bacterial cells by inducing the accumulation of reactive oxygen species.

### Ca^2+^ and Mg^2+^ ions can interfere with the bacteriostatic effect of BsR1

Antimicrobial peptides can be influenced by various abiotic factors during their application, including high temperature, ion stress, ultraviolet (UV) radiation, and acid-base conditions. In our study, we examined the impact of these factors on the antibacterial activity of the antimicrobial peptide BsR1 by subjecting it to different abiotic stresses and using it to treat four test strains. Our findings reveal that BsR1 exhibits remarkable UV stability and thermal stability (Fig. S3b and c), as it maintains strong antibacterial activity even after 2 hours of UV treatment and exposure to high temperatures up to 100°C. In terms of acid-base tolerance, only the antibacterial activity against *Xoc* was significantly affected by acid-base stress (Fig. S3a), while the activity against other strains remained unaffected.

**Fig 3:**
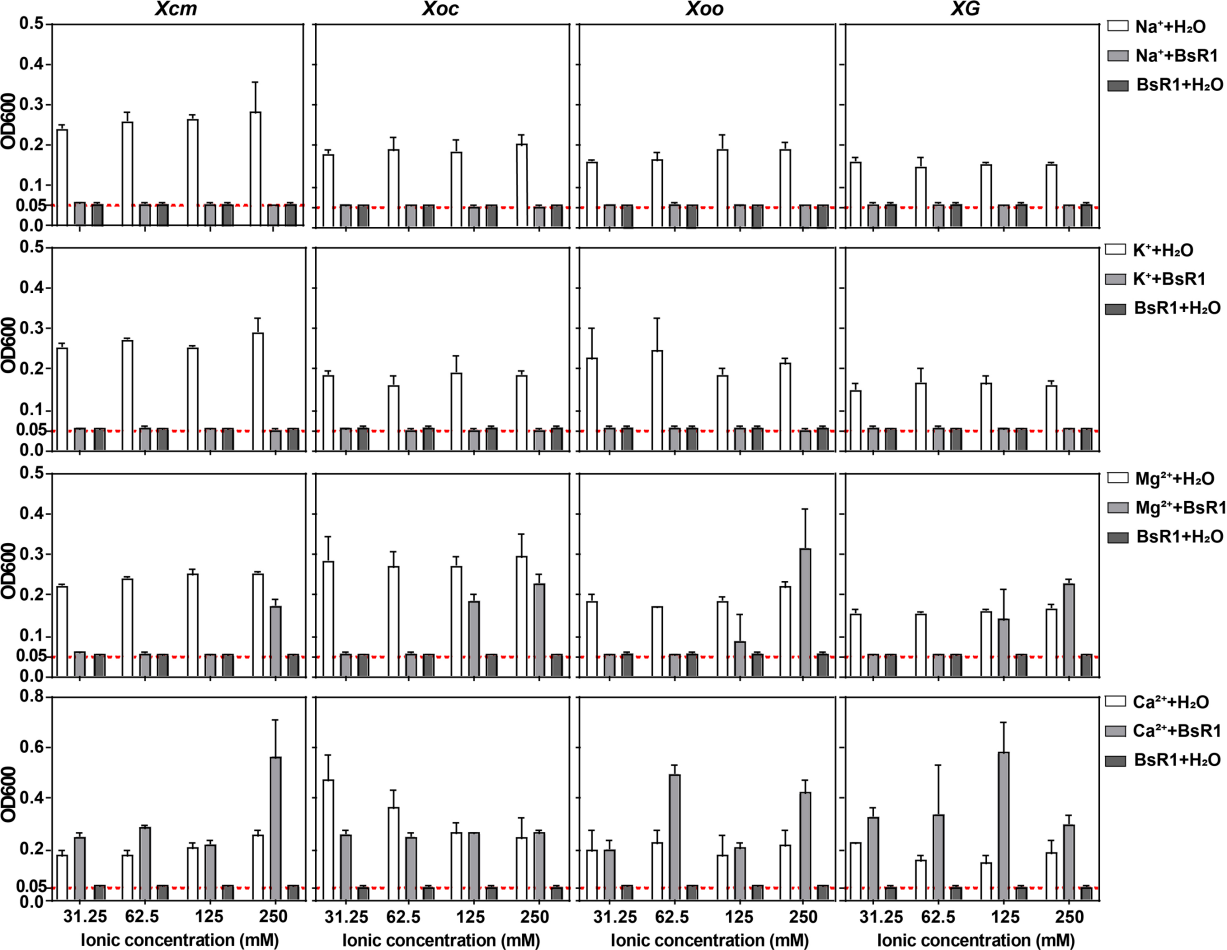
Ionic stability determination of the antimicrobial peptide BsR1. The experiment involved testing the stability of BsR1 in the presence of monovalent cations Na^+^ and K^+^, as well as divalent cations Mg^2+^ and Ca^2+^. BsR1 was mixed with various concentrations of ionic solutions for 30 minutes before treating the tested strains. The negative control consisted of a mixture of ddH_2_O and ionic solutions, while the positive control consisted of a mixture of BsR1 and ddH_2_O. The red dotted line indicating OD_600_ = 0.05 served as the standard line, and concentrations near or below this line were considered inhibitory. Each experiment was independently repeated three times with three replicates each time. The error bar represents the standard deviation (SD).

Interestingly, we observed that the antibacterial activity of BsR1 was highly sensitive to divalent cations. To investigate this further, we examined the effect of different concentrations of monovalent cations (Na^+^ and K^+^) and divalent cations (Mg^2+^ and Ca^2+^) mixed with BsR1 on the four tested strains. The concentrations tested were 31.25 mM, 62.5 mM, 125 mM, and 250 mM. As shown in Fig. 3, the monovalent cations did not affect the antibacterial activity of BsR1, even at the highest concentration tested (250 mM). However, the presence of Mg^2+^ had a significant disruptive effect on the antibacterial activity of BsR1 against *Xcm* at 250 mM, nearly abolishing its activity. For *Xoc*, *Xoo*, and *XG*, the antibacterial activity of BsR1 was lost when the Mg^2+^ concentration reached 125 mM. In contrast, BsR1 exhibited high sensitivity to Ca^2+^. Even at the lowest test concentration (31.25 mM), the OD_600_ values of the tested strains were well above 0.05, indicating the loss of antibacterial activity by BsR1.

Previous research has indicated that divalent cations such as Mg^2+^ and Ca^2+^ preferentially bind to negatively charged bacterial cell membranes when present with antimicrobial peptides in the bacterial fluid. This implies that antimicrobial peptides can only bind to cell membranes by displacing these cations, which is necessary to exert their antibacterial effect. Conversely, the competitive binding of divalent cations and antimicrobial peptides is also a mechanism that mediates bacterial resistance to antimicrobial peptides. Our study provides evidence that monovalent cations do not interfere with the activity of antimicrobial peptides, while high concentrations of divalent cations, such as Mg^2+^ and Ca^2+^, antagonize their effectiveness.

### Identification of the bactericidal effect of BsR1

Antimicrobial peptides primarily act on the cell membrane, disrupting its structure and leading to the leakage of cellular contents and subsequent cell death. To assess cell death, staining techniques involving small molecular substances like propidium iodide (PI) can be employed. PI is unable to penetrate the intact cell membrane of living cells but can enter the membranes of dead cells, binding to DNA and emitting a red fluorescent signal upon excitation. Flow cytometry (FCM) can then be utilized to excite each passing cell with an appropriate laser wavelength, measuring the fluorescence intensity and number of cells exhibiting specific fluorescence intensity levels to determine the death status of treated cells.

**Fig 4:**
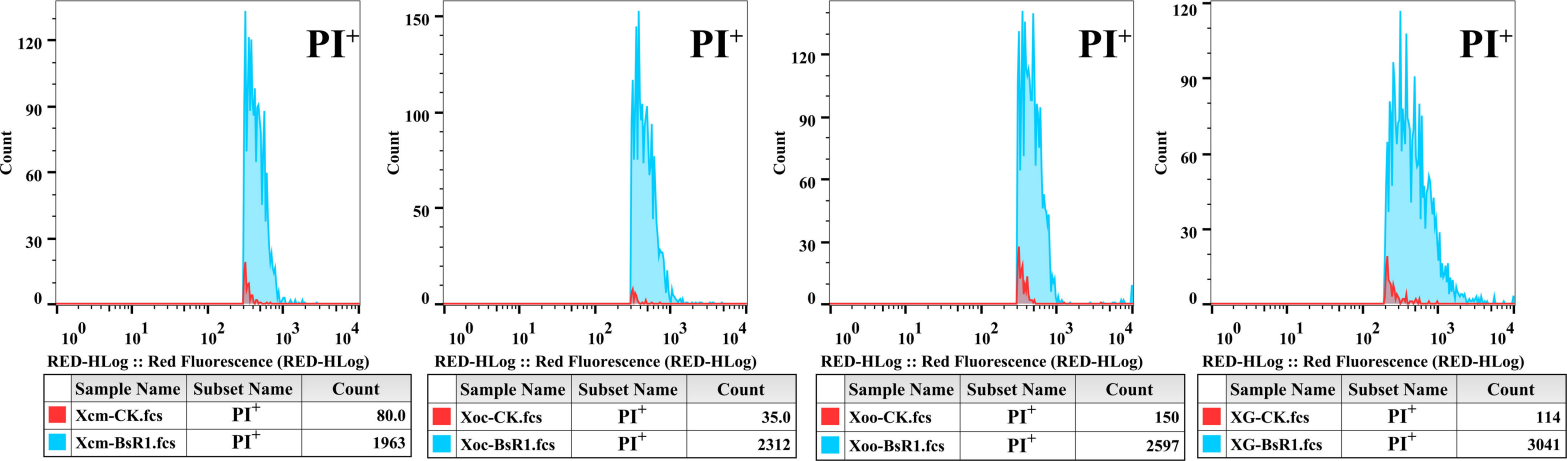
Statistical analysis of bacterial cell death following antimicrobial peptide treatment. Bacterial cells were treated with BsR1 at a concentration of 1× MIC for 1 hour and subsequently stained with propidium iodide (PI). The amount of red fluorescence in the cells was detected and quantified using a Guava easyCyte8 flow cytometer. The x-axis represents the red fluorescence intensity, while the y-axis represents the number of cells corresponding to each fluorescence intensity. The histograms are superimposed on the red fluorescence positive results of the control and treatment groups. The blue area represents the number of PI^+^ cells after BsR1 treatment, while the red area represents the number of PI^+^ cells after sterile water treatment. Five thousand cells were tested in each group, and each set of experiments was repeated three times independently.

In our study, the four tested bacterial cell types were treated with BsR1 at a concentration of 1× MIC for 1 hour and stained with PI, and cell membrane compromised situation was determined using flow cytometry. The staining results of bacterial cells treated with BsR1 for 1 hour were analyzed. The histograms presented in Fig. 4 depict the PI staining results after 1-hour treatment of bacterial cells with the negative control (ddH_2_O). The proportion of compromised bacterial cells (PI^+^) significantly increased following treatment with BsR1. For *Xcm*, the percentage increased from 1.6% to 39.3%; for *Xoc*, it increased from 0.7% to 46.2%; for *Xoo*, it increased from 3% to 51.9%; and for *XG*, it increased from 2.28% to 60.8%. The criterion for determining the number of compromised cells in the treatment group is based on at least 95% of the cells in the negative control group being red fluorescence negative (PI^−^), representing living cells. Accordingly, the number of cells that were PI^+^ after BsR1 treatment was significantly increased compared to the negative control, indicating a significant elevation in cellular mortality. This suggests that BsR1 treatment enhances the cell membrane compromised rate of bacterial cells.

### Study on the antibacterial mechanism of BsR1

The mechanism of action of BsR1 is intriguing due to its remarkable ability to rapidly inhibit bacteria and increase the cell membrane compromised rate of bacterial cells. Typically, antimicrobial peptides employ a membrane damage mechanism, and BsR1 was initially investigated for its membrane damage mechanism by examining cell damage through scanning electron microscopy (SEM) and confocal microscopy.

In the SEM analysis, three test strains were treated with BsR1 at a concentration of 1× MIC for 1 hour, while sterile water served as the negative control. The SEM results revealed that the strain treated with sterile water displayed a smooth surface, intact cell structure, and no signs of damage. In contrast, the *Xoc* cells treated with BsR1 exhibited severe damage, with numerous cell fragments remaining on the bacterial surface and irregularly damaged areas. Similarly, the *Xoo* and *XG* cells showed incomplete structures with cracks, detached cell walls/cell membranes, shrinkage, deflation, and partial collapse. The SEM observations indicated that BsR1 inflicted irreversible damage to the bacterial cell structure, resulting in cell damage, leakage of cellular contents, and even separation of the cell wall/cell membrane from the bacteria.

**Fig 5:**
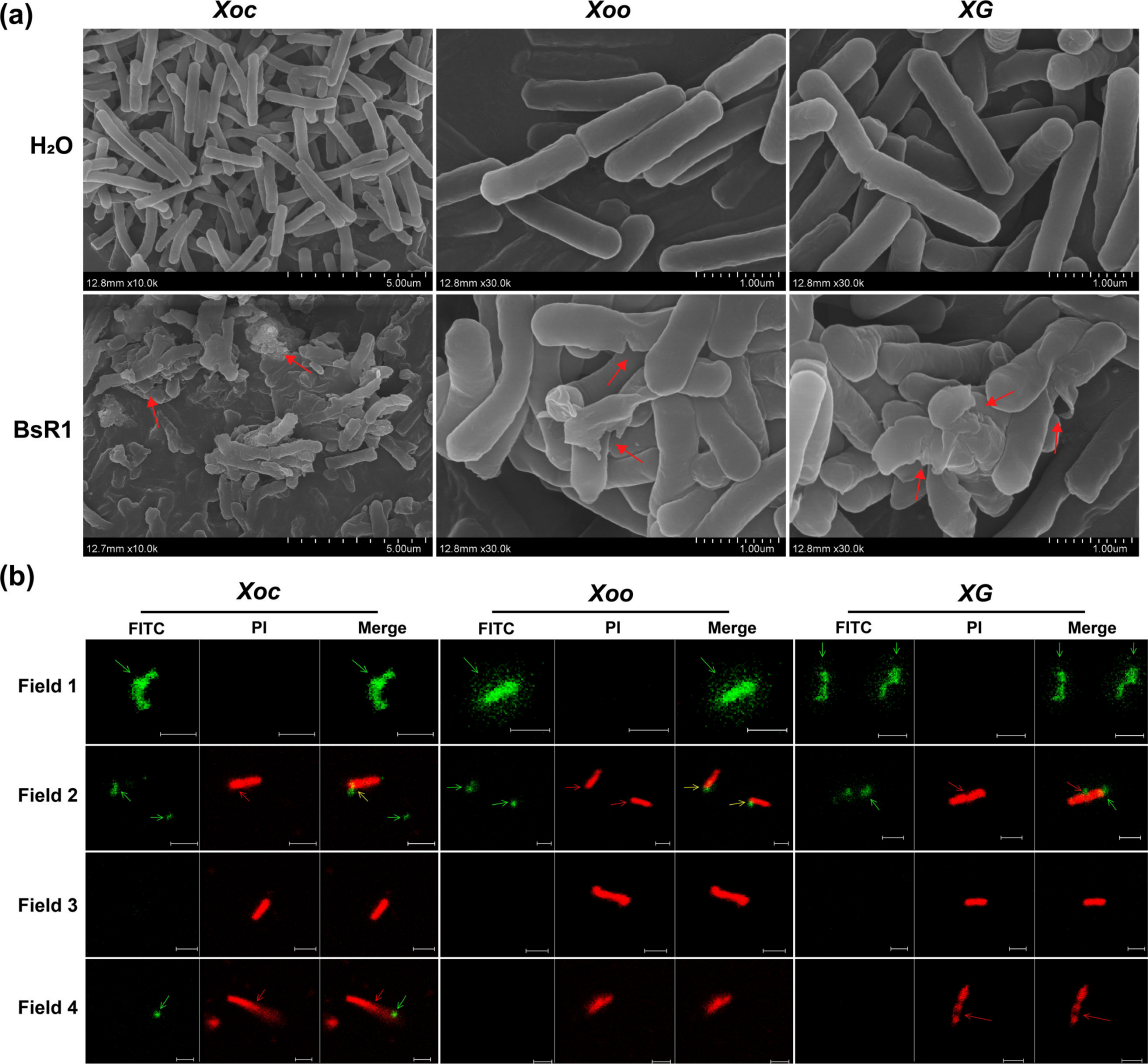
Antibacterial mechanism of BsR1. (a) Scanning electron microscopy was used to observe the destructive effect of the antimicrobial peptide BsR1 on bacterial cell structure. Cells were treated with BsR1 at a concentration of 1× MIC for 1 hour, while ddH_2_O treatment served as the negative control. The images show the cell morphology, with intact and smooth cell surfaces observed in the ddH_2_O-treated cells. In contrast, the BsR1-treated cells exhibited severe damage, including disrupted cell structures and detachment of the cell wall/cell membrane. The broken cells and damaged sites are indicated by red arrows. The scale bars represent 5 µm and 1 µm, respectively. (b) The disruptive effect of BsR1 on bacterial cell structure was observed using Annexin V-FITC/PI staining. The stained cells were visualized in four states using a dual-channel laser confocal microscope. Green fluorescence represents the Annexin V-FITC and phosphatidylserine combination, while red fluorescence indicates the PI and DNA combination. The left panel shows the FITC channel, the middle panel shows the PI channel, and the right panel shows the overlay of both channels. The bar is set at 2 µm. The green arrow indicates the Annexin V-FITC-PS complex, the red arrow indicates the PI-DNA complex, and the yellow arrow indicates the FITC/PI co-localization signal.

Confocal microscopy was employed to investigate the impact of BsR1 on bacterial cells. Following treatment with BsR1 at a concentration of 1× MIC for 1 hour, Annexin V-FITC/PI co-stained bacterial cells were examined. Phosphatidylserine (PS) is located on the cell membrane of bacterial cells, and Annexin V specifically recognizes and binds to PS. FITC (fluorescein isothiocyanate isomer) was used to label Annexin V, which emitted green light upon excitation. PI was utilized to bind DNA and emit red light upon excitation. By simultaneously staining the cells with Annexin V-FITC and PI, the state of the cell membrane and cell death could be observed simultaneously (as depicted in Fig. 5b). Four cell states (type 1–4) were observed in all three types of cells.

Type 1 cells exhibited a green fluorescent signal in the FITC channel, indicating the binding of Annexin V to PS on the cell membrane. In *Xoo* and *XG* cells, the FITC-labeled Annexin V-PS complex detached from the cell structure and dispersed (as indicated by the green arrow). However, no red fluorescent signal from PI was observed, indicating that the bacteria were not yet dead. Type 2 cells displayed fluorescent signals in both the FITC and PI channels, with a clear outline of the cell structure. The Annexin V-PS complex had not completely separated from the cell structure, indicating its presence inside the cell. Meanwhile, the cell exhibited a co-localized yellow signal indicating the presence of PI. This state represented a transition in which the membrane substances represented by the Annexin V-PS complex had not fully detached from the cell structure, but the bacteria were already dead. Type 3 cells showed no fluorescent signal in the FITC channel, but the red fluorescent signal from PI-DNA remained inside the cell. This indicated that the membrane substances represented by the Annexin V-PS complex had completely separated from the cell structure or bacteria. Although the cell membrane had not inverted, it had lost its activity, resulting in the direct death of the bacteria. Type 4 cells displayed disintegration and dispersal of the cell structure marked by FITC and PI, leading to the release of cellular contents and cell breakage and death.

The combination of Fig. 5a and b clearly illustrated that BsR1 caused the destruction of the cell membrane/cell wall, leading to detachment, lysis of the cell, release of cellular contents, and, ultimately, cell death. This strongly supports the notion that BsR1 operates through a classical membrane damage mechanism. Furthermore, the results presented in Fig. 2d indicated that BsR1 could induce bacterial oxidative stress within a short period, further corroborating the membrane damage mechanism of action.

### Biosafety and disease resistance of BsR1

**Fig 6:**
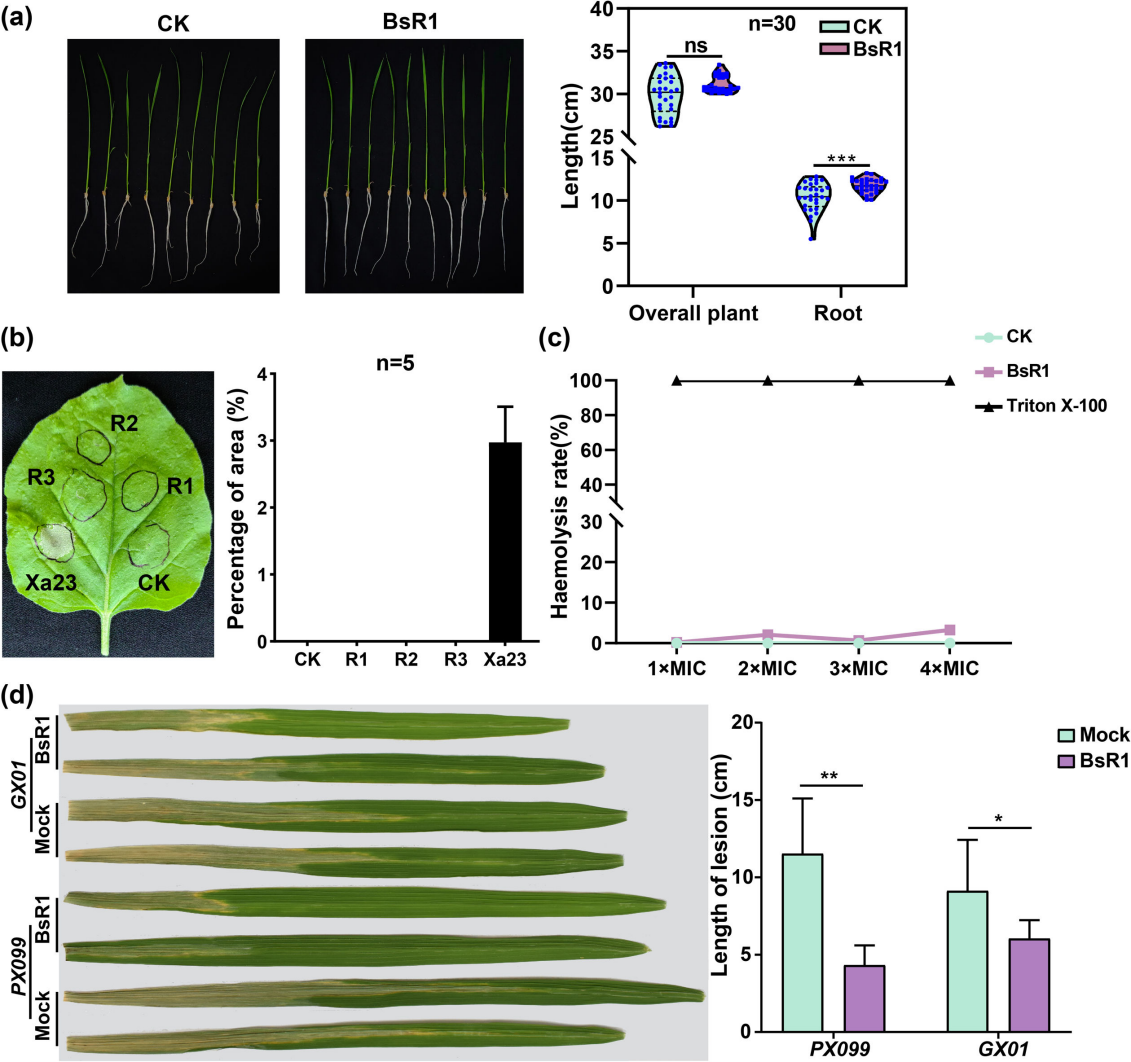
Biosafety and disease resistance of BsR1. (a) The impact of the antimicrobial peptide BsR1 on rice seedling growth was assessed, and no inhibitory effect was observed. The negative control used ddH_2_O, and the concentration of BsR1 was 1× MIC. The analysis was performed using an unpaired *t*-test, with a sample size of *n* = 30 rice seedlings. The blue dots represent data for each rice length. (b) The expression of BsR1 in tobacco cells was found to be non-harmful. The circle shown by the black line represents the injection area. Four different conditions were tested: P1300 as the negative control (CK), R1 containing the *BsR1-Flag-GFP* fusion gene, R2 containing the *BsR1-Flag* fusion gene, and R3 expressing *BsR1* alone. Additionally, the effect of the rice bacterial blight resistance gene, *Xa23*, known for inducing necrosis in tobacco leaves, was evaluated. The sample size was *n* = 5. (c) The hemolytic activity of BsR1 was assessed using sheep blood cells. Different MIC concentrations of BsR1 were tested over a 2-hour period, with normal saline as the negative control and 1% Triton X-100 as the positive control. The OD_540_ values were measured using a microplate reader. The different colored lines represent the rate of hemolysis after treatment. (d) The inhibitory effect of BsR1 on the expansion of rice bacterial blight spots was investigated using two physiological races of rice bacterial blight (PX099 and GX01) as test strains. Rice flag leaves were inoculated with a mixed bacterial solution containing BsR1 at a final concentration of 68 µM, while sterile water served as the negative control. The data are presented in the form of histogram, the analysis utilized an unpaired *t*-test, with *n* = 5 rice pots tested. The error bar represents the standard deviation (SD).

The safety evaluation of antimicrobial peptides is a critical concern due to their unique mechanism of action. In our study, we thoroughly investigated the toxicity of BsR1 on both plant and animal cells. To assess the plant safety of antimicrobial peptides, we conducted experiments using rice and tobacco as model plants. Germinated rice seeds were treated with a hydroponic solution containing BsR1 at a final concentration of 34 µM in the treatment group, while the control group received an equal volume of sterile water. After 2 weeks of cultivation, we measured the total length and root length of 30 rice seedlings (as depicted in Fig. 6a). The total length of rice seedlings treated with BsR1 showed a slight increase compared to the control group, but the difference was not statistically significant. However, the root length exhibited a significant increase after BsR1 treatment. These findings suggest that antimicrobial peptides have a limited growth-promoting effect on rice roots during the developmental stage of seedlings, while their impact on the overall length of rice is not substantial.

In the case of tobacco leaves, different expression vectors were employed, including a positive control (Xa23 expression vector) and a negative control (P1300 empty expression vector). The results indicated that only the positive control displayed necrotic spots, whereas the leaves injected with antimicrobial peptide expression vectors (R1, R2, and R3) and the negative control remained healthy. The proportion of the necrotic area in tobacco leaves was approximately 3% for the positive control, suggesting that antimicrobial peptides had no toxic effect on tobacco leaf cells.

To assess the safety of antimicrobial peptides on animal cells, we used sheep red blood cells as the research subject. The sheep red blood cell suspension was treated with different concentrations of BsR1 (1× MIC, 2× MIC, 3× MIC, 4× MIC), along with normal saline as the negative control and 1% Triton X-100 as the positive control. After a 2-hour treatment at 37°C, the OD value at 540 nm (OD_540_) of the supernatant solution was measured, and the hemolysis rate was calculated using the following formula: $Hemolysis\;rate\;(\%)=\frac{OD540_{BsR1}-OD540_{CK}}{OD540_{Triton}-OD540_{CK}}\times \;100\%$ . The results demonstrated that even at a concentration of 4× MIC, BsR1 exhibited a low hemolysis rate (3.26%) on sheep red blood cells. At a concentration of 1× MIC, the hemolysis rate was only 0.2%, indicating that BsR1 is relatively safe for animal cells at the working concentration.

For the investigation of disease resistance, we selected Kitaake as the inoculation target and used two bacterial blight races (PX099 and GX01) to inoculate rice. The control group received a bacterial solution with sterile water, while the treatment group received a bacterial solution with BsR1. After 21 days of inoculating rice flag leaves using the leaf-cutting method, the incidence of rice leaf disease and the length of the lesions were recorded. The results demonstrated that the treatment group supplemented with BsR1 significantly reduced the disease incidence, and the lesion length of the leaves was considerably smaller than that of the control group. This indicates that BsR1 can effectively inhibit the expansion of rice bacterial blight to a certain extent. These results are illustrated in Fig. 6c and d, respectively.

## DISCUSSION

This study presents a significant achievement in the prediction and optimization of the active region of the antifungal gene *Fg*Mt1, leading to the discovery of a novel cationic antibacterial peptide, BsR1. BsR1 exhibited a remarkable spectrum of antibacterial activity with a low MIC value, as low as 4.25 µM, and demonstrated notable efficacy against *Xanthomonas* bacteria. The peptide also demonstrated excellent stability under various conditions, including thermal, UV, pH, and monovalent ion stability. However, the antibacterial activity of BsR1 was found to be sensitive to divalent cations, such as Ca^2+^ and Mg^2+^, as high concentrations of these ions inhibited its activity. This observation is consistent with the crucial role divalent cations play in stabilizing the bacterial membrane structure. It is well established that many antimicrobial peptides lose their activity in the presence of high concentrations of divalent cations (50). The outer membrane of Gram-negative bacteria is primarily composed of lipopolysaccharide (LPS), and the integrity of LPS is stabilized by divalent cations (51, 52). Specifically, antimicrobial peptide magainin 2 competes with Mg^2+^ for binding to LPS, disrupting the homeostasis of LPS on the cell membrane. The integrity of LPS is restored as the concentration of Mg^2+^ increases (53). Consequently, high concentrations of Mg^2+^ can stabilize the LPS layer, impeding the binding of antimicrobial peptides to LPS (54).

These findings highlight the role of divalent cations in hindering the effectiveness of antimicrobial peptides by disrupting the interaction between peptides and the lipid bilayer. Given that BsR1 targets the cell membrane and is inhibited by divalent cations, it is necessary to address this issue to enhance its efficacy. One potential strategy is to increase the overall hydrophobicity of antimicrobial peptides by substituting bulky nonpolar amino acids, as this can potentially augment the peptide’s interaction with the lipid bilayer (55). Additionally, incorporating end-coupling hydrophobic moieties can further enhance hydrophobic interactions with the bacterial membrane (56). Moreover, efforts should be made to reduce the salt sensitivity of BsR1. By implementing these improvements, the effectiveness of BsR1 and other antimicrobial peptides can be enhanced. This discussion provides valuable insights into the potential limitations of antimicrobial peptides and proposes practical approaches to overcome these challenges, ultimately improving their efficacy.

Phosphatidylserine (PS) plays a critical role in bacterial membranes (57). These membranes exhibit asymmetry, with PS primarily located in the inner leaflet of the plasma membrane. Disruption of membrane fluidity can lead to the loss of asymmetry, causing PS to flip from the inner to the outer leaflet during apoptosis. Many antimicrobial peptides exert their effects by damaging the cell membrane, and the distribution of PS can serve as an indicator of membrane status. Annexin V is an annexin protein that binds to PS-containing membranes in the presence of Ca^2+^ and is recruited following membrane damage (58). The use of FITC-labeled Annexin V enables visualization of the bacterial cell membrane after treatment with antimicrobial peptides. Scanning electron microscopy and confocal microscopy results clearly demonstrate that treatment with BsR1 causes the separation of the cell wall/cell membrane from the bacteria and disrupts the asymmetric distribution of membrane lipids, as evidenced by the Annexin V-PS complex, resulting in loss of membrane fluidity. These findings indicate that BsR1 follows a classical membrane damage mechanism, as depicted in Fig. 5. Although the specific target site of BsR1 on the bacterial membrane remains unknown, it is hypothesized that anionic substances such as LPS and lipoteichoic acid may interact with BsR1. The secondary structure prediction of BsR1 also suggests its potential incorporation into the cell membrane, disrupting membrane fluidity. Furthermore, the observed interference of divalent cations with BsR1 suggests a potential competition between the two, compromising the stability of the LPS layer. However, the detrimental effects caused by BsR1 can be mitigated by divalent cations. While membrane damage is likely the primary mode of action for BsR1, it is important to acknowledge that other mechanisms may also be involved in its antibacterial activity.

Antimicrobial peptides have garnered significant attention in agricultural research; however, several pressing issues require immediate attention. First, there is a scarcity of natural antimicrobial peptides in plants, with only 371 plant-derived peptides recorded in the APD3, accounting for less than 10% of the total registered antimicrobial peptides. Second, natural antimicrobial peptides exhibit high cytotoxicity, low stability, and susceptibility to degradation, raising concerns regarding their biosecurity. Third, there is a dearth of high-throughput screening and validation methods for antimicrobial peptides. Fourth, obtaining pure antimicrobial peptides is challenging, and the synthesis process is expensive, rendering it economically unviable. Lastly, the utilization of transgenic approaches for applying antimicrobial peptides is presently not approved by authorities and may face resistance from consumers (59).

To address these challenges, we propose several potential solutions. First, we suggest the construction of small fragment DNA expression libraries from diverse species. This can be achieved by selecting species with relatively simple genomes, such as bacteria and fungi, and employing restriction nucleases or ultrasonic disruption to generate a large number of DNA fragments. These fragments can be subsequently incorporated into microbial expression vectors and screened using microbial expression systems, such as the yeast expression system (60, 61) and the *B. subtilis* expression system (62). Second, we recommend the integration of bioinformatics tools and genome databases like Ensembl to predict regions within fragments that may possess antimicrobial properties. This approach can aid in identifying potential candidates for further investigation. Third, we propose the modification and transformation of existing natural antimicrobial peptides to enhance their stability and reduce their hemolytic properties (63). By employing genetic engineering techniques, it is possible to optimize the characteristics of these peptides, making them more suitable for practical applications. Lastly, we suggest exploring the expression of antimicrobial peptides in plants (64). For instance, expressing cecropin A in rice can enhance resistance to the rice blast fungus *Magnaporthe grisea* (65), while introducing human LL-37 into transgenic barley can confer long-term stability (66). Other examples include the incorporation of the pharmaceutically active polypeptide SMAP-29 (67) and the transformation of citrus with *sarcotoxin IA*, rendering them less susceptible to *Candidatus Liberibacter asiaticus* (CLas) (68). By pursuing these strategies, we can potentially overcome the limitations associated with antimicrobial peptides and pave the way for their effective utilization in agriculture.

Plant diseases pose a significant threat to global agriculture, resulting in substantial economic losses. In the fight against these diseases, the combination of antimicrobial peptides and plant transgenic technology offers a promising and effective approach. Notably, the structural differences between bacterial cell walls and plant cell walls impact the anionic nature of bacterial cell walls, which is a crucial factor influencing the recognition of antimicrobial peptides. Consequently, many antimicrobial peptides demonstrate safety for both animal and plant cells, underscoring their importance in plant applications. Furthermore, the amino acid composition of antimicrobial peptides ensures that their degradation products do not give rise to new harmful substances, making their use in agricultural disease control safer compared to traditional antibiotics. Advancements in transgenic technology have opened doors for introducing antimicrobial peptides into plants as antibacterial genes, initially targeting non-edible crops such as cotton and tobacco. Antimicrobial peptides possess a unique mode of action that makes it challenging for microorganisms to develop drug resistance, thus slowing down the emergence of drug-resistant bacteria. However, this approach presents several challenges that need to be addressed. These include formulating effective expression strategies, identifying suitable transgenic plants, determining optimal levels of antimicrobial peptide expression, and ensuring the stability of transgenic plants throughout their growth. While these challenges exist, they are accompanied by numerous opportunities on this transformative journey. It is our aspiration that antimicrobial peptides will evolve into a potent weapon for effectively controlling plant diseases in the future, ultimately contributing to sustainable and resilient agriculture.

## MATERIALS AND METHODS

### Bacterial material, plant material, and culture environment

The research utilized several strains including *Bacillus* sp. A, *Bacillus* sp. K1, *Clavibacter fangii*, *Clavibater michiganensis* , *Rastonia solanacearum* (R21-5), *Xanthomonas campestris* pv. *Holcicola* (*Xcm*), *Xanthomonas oryzae* pv. *oryzicola* (*Xoc*), and *Xanthomonas oryzae* pv. *oryzae* (race 4, *XG*), and *Xanthomonas oryzae* pv. *oryzae* (*Xoo*). *Bacillus* sp. A and *Bacillus* sp. K1 were cultured in LB medium for 10 hours at 28°C and 170 rpm to harvest the bacterial cells.

### Strain activation and culture process

To activate the strains, *Xcm*, *Xoc*, *Xoo*, and *XG* are streaked into an ordinary LB plate using an inoculation loop. The plate is then cultured overnight at 28°C, and a single colony grown on the plate is picked and placed in the liquid LB medium. The culture is shaken for about 10 hours at 170 rpm, and the OD_600_ value of the bacterial solution is measured every 30 minutes until the shake culture reaches the logarithmic growth phase (OD_600_ = 0.8–1.0).

The Kitaake material is used for rice, and the *Nicotiana benthamiana* material is used for tobacco. The pathogenic races PX099 and GX01 of *Xanthomonas oryzae* are used for inoculating rice. The vectors used for transforming tobacco with Agrobacterium are *pSuper-1300-GFP*, R1 is *pSuper-1300-BsR1-GFP-Flag*, R2 is *pSuper-1300-BsR1-Flag*, R3 is *pSuper-1300-BsR1*, and Xa23 is *pSuper-1300-Xa23-GFP*.

### Plate inhibition assay

Determination of bacterial inhibitory activity of antimicrobial peptides by plate inhibition method: various bacterial strains, including *Bacillus* sp. A, *Bacillus* sp. K1, *C. fangii*, *C. michiganensis*, *R. solanacearum*, *X. campestris* pv. *Holcicola* (Xcm), *X. oryzae* pv. *oryzicola* (Xoc), *X. oryzae* pv. *oryzae* (race 4, XG), and *X. oryzae* pv. *oryzae* (Xoo), were subjected to specific incubation conditions. *Bacillus* strains were incubated at 37°C with shaking, while all other strains were incubated at 28°C with a shaking speed of 170 rpm for 12–14 hours. Following incubation, 200 µL of the bacterial fluids was transferred to 10-mL shaking tubes, mixed with 4 mL of semi-solid LB medium at approximately 50°C. The resulting mixture was promptly shaken and poured onto a solid LB plate, ensuring even distribution by gentle shaking. The plated semi-solid LB medium was allowed to dry and solidify for approximately 5 minutes. Subsequently, the petri dish was divided into four quadrants, each containing a sterile filter paper sheet. For the antimicrobial assay, 20 µL of the antimicrobial peptide, along with negative and positive controls, was carefully aspirated and slowly dripped onto the filter paper in one quadrant. This process facilitated the slow and even spread of the liquid. The petri dish was then placed in the incubator at 37/28°C for 5–7 hours. Observations were made periodically to determine the appearance of a ring of inhibition.

### MIC and MBC measurement

In comparison with previous literature (49 – 53), the method for determining MIC has been modified. For bacterial culture, refer to the Strain activation and culture process. Bacterial solutions were appropriately diluted using LB to attain an OD_600_ falling within the range of 0.02–0.05. Subsequently, 80 µL of this bacterial solution was aliquoted into a 96-well board (Thermo Scientific, AB0600). Concurrently, the antimicrobial peptide BsR1 was diluted to various concentrations (340 µM, 170 µM, 85 µM, 42.5 µM, and 21.25 µM) through the establishment of a concentration gradient, with a total volume of 20 µL. These diluted BsR1 solutions were then combined with the 80 µL of bacterial solution, resulting in final BsR1 concentrations of 68 µM, 34 µM, 17 µM, 8.5 µM, and 4.25 µM, respectively. Following thorough mixing, the composite solution was incubated in a 28 ℃ incubator for 24 hours, after which the OD_600_ value was quantified using a microplate reader. As a positive control, polymyxin B was employed (0.2 mg/mL).

To determine the MBC, a mixed solution of antibacterial peptides and bacteria solution was cultured for 24 hours. Afterward, 100 µL of the mixture was spread on LB plates and incubated at 28°C overnight. The number of single colonies on each plate was recorded, and the minimum concentration of antimicrobial peptide that resulted in less than or equal to five single colonies per plate was considered the MBC value.

### ROS measurement

For cell culture, refer to the Strain activation and culture process. Following the centrifugation of cells at 664 *× g* for 5 minutes at 4℃, the cells were resuspended in a 20 mM PBS (phosphate buffered saline) buffer. The resuspended cells were subsequently introduced into a 96-well plate (Thermo Scientific, AB0600). Varied concentrations of BsR1, constituting the treatment group, were mixed with sterile water, representing the control group. This mixture included 10 µg/mL horseradish peroxidase and 50 µM luminol. The resulting combination was then added to the bacterial solution and promptly placed into a microplate reader. The OD_425_ value in the medium was detected, and the fluorescence intensity was recorded over time. It is noteworthy that this methodology draws inspiration from the work of Choi et al., with certain modifications (69).

### BsR1 stability measurement

For cell culture, refer to the Strain activation and culture process. In order to investigate the stability of antimicrobial peptide BsR1, various experiments were conducted. For thermal stability, the peptide was treated at temperatures ranging from 4°C to 100°C for 30 minutes each, with the control being the treatment at 4°C. After cooling to room temperature, the peptide was added to a 96-well plate containing bacterial solution at a final concentration of 1× MIC. For UV stability, the peptide and sterile water were exposed to UV light for varying durations ranging from 0 to 120 minutes, with the control being the exposure for 0 minute. The final concentration was 1× MIC. To test pH stability, the peptide was mixed with solutions of different pH levels (ranging from 3 to 10) prepared using 5 M NaOH and 5 M HCl. After 30 minutes of treatment, the peptide was added to the bacterial solution in the 96-well plate at a final concentration of 1× MIC. A positive control was prepared by mixing sterile water and BsR1, while a negative control was prepared by mixing sterile water and different pH solutions. Finally, to test the ion stability, the peptide was mixed with solutions of different concentrations of NaCl, KCl, MgSO_4_, and CaCl_2_ (ranging from 31.25 mM to 250 mM) and treated for 30 minutes. The mixture was then added to the bacterial solution in the 96-well plate. Sterile water mixed with BsR1 was used as a positive control, and sterile water mixed with different concentrations of ionic solutions was used as a negative control with a final concentration of 1× MIC.

### FCM for cell mortality

Cell death was measured using flow cytometry (Guava easyCyte8, Millipore) (70, 71). For cell culture, please refer to Strain activation and culture process. The cells were collected through centrifugation at 664 *× g* for 5 minutes at 4°C and resuspended in 20 mM PBS buffer, and BsR1 was added to achieve a final concentration of 1× MIC concentration. The cells were then treated for 1 hour, with sterile water serving as the negative control. After treatment, the cells were centrifuged at 664 *× g* for 5 minutes, washed with PBS twice, and resuspended. PI was added to achieve a final concentration of 20 µg/mL, and the cells were stained for 10 minutes before being detected by flow cytometry with an excitation wavelength of 488 nm. The cellular mortality rate was calculated according to the following: number (PI^+^)/number (cell count).

### SEM and confocal laser scanning microscope (CLSM)

To initiate cell culture, the procedure outlined in Strain activation and culture process was followed. Post-collection of cells via centrifugation at 664 *× g* for 5 minutes at 4°C, they were resuspended in a 20 mM PBS buffer. Subsequently, BsR1 was added to achieve a final concentration of 1× MIC concentration, with aseptic water employed as a negative control. This treatment endured for 1 hour. Following treatment, a centrifugation step at 664 *× g* for 5 minutes facilitated cell collection, and cells were washed twice with PBS. The ensuing steps involved resuspending the cells in 2.5% glutaraldehyde for fixation over a 4-hour period. After another round of centrifugation at 664 *× g* for 5 minutes, the cells underwent successive washing steps with 30%, 50%, 70%, 90%, and 100% ethanol. The cells were once more collected via centrifugation at 664 *× g* for 10 minutes. Subsequent to air-drying the sample in a fume hood for 10 minutes, it underwent freeze drying to transform into a powder. Finally, the sample was scrutinized using a JEOL JSM-7001F scanning electron microscope (72, 73).

For cell culture, refer to Strain activation and culture process. Following the appropriate time frame for treatment, cells are to be gathered via centrifugation at 664 *× g* for 5 minutes at 4°C. The subsequent step involves resuspending the cells in a 20 mM PBS buffer. BsR1 is then introduced to attain a final concentration equivalent to 1× MIC. This treated state should persist for 1 hour, utilizing aseptic water as the negative control. Post-treatment, the cells are to undergo centrifugation at 664 *× g* for 5 minutes, followed by two washes with PBS. The final step involves resuspending the cells for further analysis. Staining of cells was performed using the Annexin V-PI Apoptosis Detection Kit (Bestbio) (74, 75), and the results were observed using CLSM (Leica microsystems CMS GmbH TCS SP8; Leica, Germany).

### Hemolytic activity

To prepare a 4% sheep red blood cell suspension, sterile defibrinated sheep blood is taken, and it is centrifuged at 3000 rpm for 5 minutes. Following centrifugation, the blood is washed with 0.9% normal saline in a repetitive cycle of three times until the supernatant achieves clarity and transparency. The lower layer, consisting of red blood cells, is then separated, and a 4% erythrocyte suspension is formulated with normal saline, adhering to a volume ratio of V1/V2 = 4/100. BsR1 is subsequently introduced to attain final concentrations of 1× MIC, 2× MIC, 3× MIC, and 4× MIC. As part of the experimental controls, normal saline serves as the negative control, while 1% TritonX-100 is utilized as the positive control. Following a 2-hour treatment period, the supernatant is subjected to centrifugation and subsequently transferred to a 96-well plate. The measurement of the OD_540_ value for each well is performed, and the hemolysis rate of the antimicrobial peptide is calculated using the following specific formula:


$$Hemolysis\;rate\;(\%)=\frac{OD540_{BsR1}-OD540_{CK}}{OD540_{Triton}-OD540_{CK}}\times \;100\%$$

### Biosafety and disease resistance of BsR1

#### Determination of the safety of antimicrobial peptides on rice seedlings

Rice seeds (cultivar: Kitaake) were subjected to a rigorous sterilization procedure. Initially, a 5-minute treatment involving 75% ethanol was administered, followed by immersion in a 5 mg/L sodium hypochlorite solution. To eliminate any residual sodium hypochlorite, the seeds underwent three meticulous 10-minute washes. Subsequent to this sterilization process, the seeds were immersed for a duration of 2–3 days, during which daily monitoring was conducted to assess the bleaching status. Whitened seeds were judiciously selected, extracted, and allocated to individual wells within a 96-well plant culture box. This allocation followed a systematic pattern of placing one seed per alternating well. The selected seeds were submerged in water within the container, and a layer of plastic wrap was applied to sustain moisture. After a 2-day incubation period, the plastic wrap was removed. Following seedling emergence, the entire water volume was decanted, and a nutrient-rich culture solution, containing both micronutrients and macronutrients, was introduced. Simultaneously, the antimicrobial peptide BsR1 was incorporated, achieving a final concentration of 34 µM. Sterile water was employed as a control in tandem with these experimental conditions.

#### Determination of the safety of antimicrobial peptides on tobacco leaves

Concentrations of antimicrobial peptides were methodically standardized and subjected to serial dilution, resulting in concentrations of 0.034 µM, 0.34 µM, 3.4 µM, and 34 µM. Sterile water served as the diluent for preparing stock solutions. Following this, tobacco plants displaying vigorous growth at the four- to five-leaf developmental stage were discerningly chosen for experimental purposes. In a sterile environment, 500-µL volumes of varied antimicrobial peptide concentrations were withdrawn using a sterile syringe and precisely administered onto the dorsal surface of tobacco leaves. Concurrently, parallel experiments with sterile water as a negative control were conducted. The physiological condition of the treated tobacco leaves was systematically observed at 24-hour intervals subsequent to the injection procedure.

#### Determination of resistance of antimicrobial peptides to bacterial leaf blight in rice

PX099 and GX01 were carried out on LB medium at 28°C. After 2–3 days of culture, the organisms were collected for inoculation. For inoculation, dilute with sterile water to OD_600_ = 0.5. PX099 and GX01 inoculation was performed by leaf cutting, using scissors dipped in the bacterial solution to cut the tips of the newest extended rice leaves at 5 cm during the spike stage, and investigating the length of the leaf spots 14 days after inoculation.

### Bioinformatics predictions and data analysis

The N-terminator and C-terminator of the antimicrobial peptide were predicted using AntiBP Server (61), and the APD3 (https://aps.unmc.edu/home) and RPBS (https://mobyle.rpbs.univ-paris-diderot.fr/cgi-bin/portal.py#welcome) predicted the secondary structure of the antimicrobial peptide BsR1. GraphPad Prism version 8.00 (GraphPad Software, San Diego, CA, USA; https://www.graphpad.com/) was used to analyze the experimental data as well as to make graphs. The statistical analyses involved in this study were performed using Student’s *t*-test, with “*” indicating *P* ≤ 0.05, “**” indicating *P* ≤ 0.01, and *P*-values were determined using false discovery rate.

## SUPPLEMENTAL MATERIAL

Supplemental figures spectrum.02578-23-s0001.pdf Supplemental figures S1, S2, and S3.

ASM does not own the copyrights to Supplemental Material that may be linked to, or accessed through, an article. The authors have granted ASM a non-exclusive, world-wide license to publish the Supplemental Material files. Please contact the corresponding author directly for reuse.
