# Supplementary material for: BsR1, a broad-spectrum antibacterial peptide with potential for plant protection
Source: Microbiol Spectr. 2023 Nov 10;11(6):e02578-23. doi: 10.1128/spectrum.02578-23 (PMC10714738; doi:10.1128/spectrum.02578-23)
Supplement: Supplemental figures — S1, S2, and S3. [file spectrum.02578-23-s0001.pdf]

BsR1, a Broad-Spectrum Antibacterial Peptide with Potential for Plant Protection

Pei Song, Li Zhao, Li Zhu, Gan Sha, and Wubei Dong*

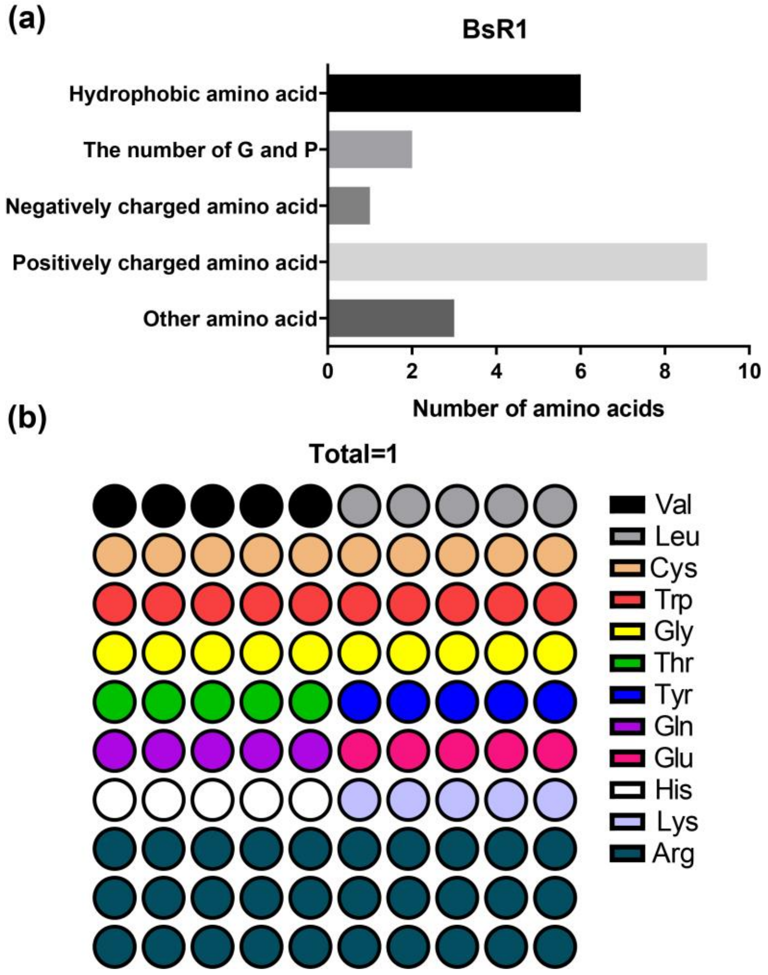

Figure S1. Amino acid composition and percentage of BsR1.

(a) The amino acid composition of BsR1 shows a relatively high content of hydrophobic and positively charged amino acids.

(b) Arginine represents the largest proportion among the amino acid composition of BsR1.

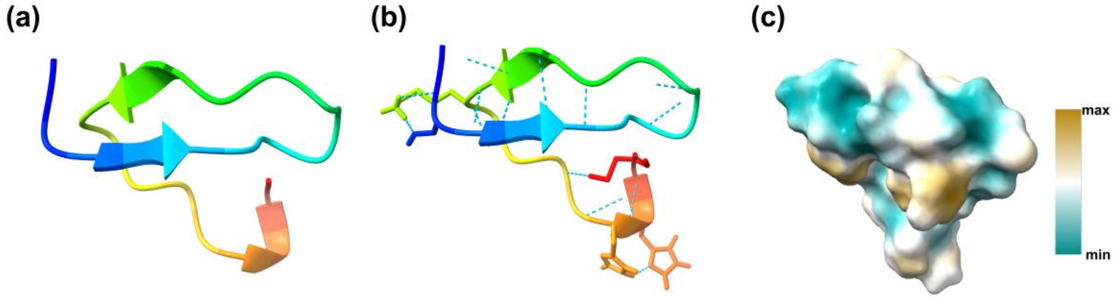

Figure S2. Secondary structure and hydrophobicity analysis of BsR2 (GSGSFYPGSASTVPVKTFLPHHRC).

(a) Predicted secondary structure of BsR2 peptide using the RPBS website. (b) Representation of the 14 potential hydrogen bonds in BsR2, indicated by blue dashed lines, as predicted by the RPBS website. (c) Hydrophobicity analysis of BsR2 surface using the RPBS website. The color scale ranges from blue to brown, indicating increasing hydrophobicity values. The structures presented above were visualized in the PDB format of BsR2 using Chimera X software.

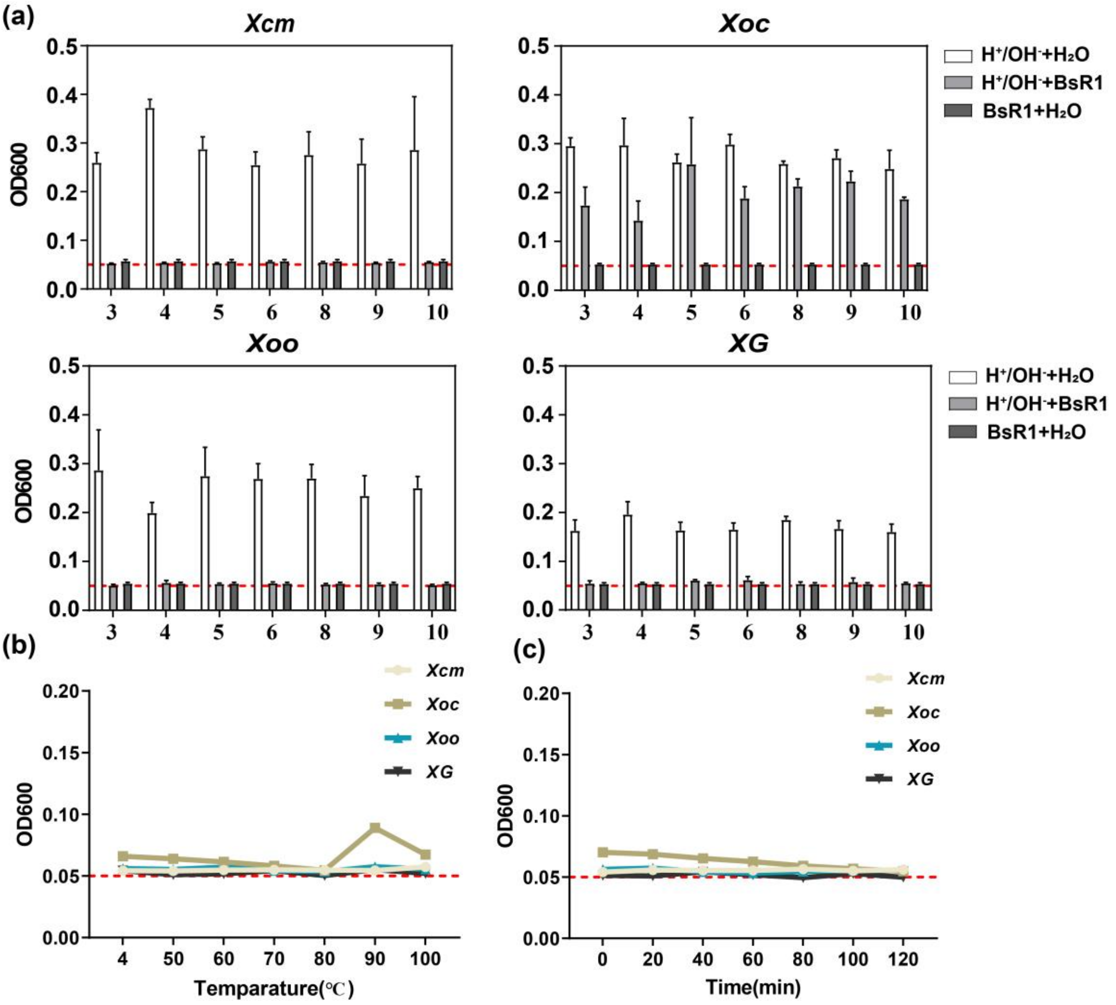

Figure S3. Stability measurements of antimicrobial peptide BsR1.

(a) Acid-base stability of BsR1. The horizontal axis represents the pH value, while the vertical axis represents OD_{600}. BsR1 was mixed with H^+/OH^- acid-base solution for 30 minutes before treating the test strain. Negative control involved mixing ddH_2O with acid-base solution, and positive control involved mixing BsR1 with ddH_2O.

(b) Thermal stability assay of BsR1. BsR1 was exposed to different temperatures for 30 minutes prior to treating the test strain, with 4°C treatment serving as the negative control.

(c) UV stability assay of BsR1. BsR1 was subjected to varying durations of UV lamp exposure before treating the test strain, with 0 minutes (no UV) treatment as the negative control. The red dashed line represents the standard line of OD_{600} = 0.05, and concentrations near or below this line are considered inhibitory. The error line indicates Standard Deviation (SD).
